# Characterization of Anchorless Human PrP With Q227X Stop Mutation Linked to Gerstmann-Sträussler-Scheinker Syndrome In Vivo and In Vitro

**DOI:** 10.1007/s12035-020-02098-8

**Published:** 2020-09-05

**Authors:** Pingping Shen, Johnny Dang, Zerui Wang, Weiguanliu Zhang, Jue Yuan, Yue Lang, Mingxuan Ding, Marcus Mitchell, Qingzhong Kong, Jiachun Feng, Annemiek J. M. Rozemuller, Li Cui, Robert B. Petersen, Wen-Quan Zou

**Affiliations:** 1 Department of Neurology, First Hospital of Jilin University, Changchun, Jilin Province People’s Republic of China; 2 Departments of Pathology and Neurology, Case Western Reserve University School of Medicine, 2085 Adelbert Road, Cleveland, OH USA; 3 National Prion Disease Pathology Surveillance Center, Case Western Reserve University, 2085 Adelbert Road, Cleveland, OH USA; 4 Dutch Surveillance Center for Prion Diseases, University Medical Center Utrecht, Utrecht, The Netherlands; 5 Foundation Sciences, Central Michigan University College of Medicine, Mount Pleasant, MI USA; 6 National Center for Regenerative Medicine, Case Western Reserve University School of Medicine, Cleveland, OH USA

**Keywords:** Prions, Prion disease, Prion protein, Mutation, Allelic origin, Glycosylphosphatidylinositol (GPI) anchor, Protein misfolding cyclic amplification (PMCA), Gerstmann-Sträussler-Scheinker (GSS) syndrome

## Abstract

Alteration in cellular prion protein (PrP^C^) localization on the cell surface through mediation of the glycosylphosphatidylinositol (GPI) anchor has been reported to dramatically affect the formation and infectivity of its pathological isoform (PrP^Sc^). A patient with Gerstmann-Sträussler-Scheinker (GSS) syndrome was previously found to have a nonsense heterozygous PrP-Q227X mutation resulting in an anchorless PrP. However, the allelic origin of this anchorless PrP^Sc^ and cellular trafficking of PrP^Q227X^ remain to be determined. Here, we show that PrP^Sc^ in the brain of this GSS patient is mainly composed of the mutant but not wild-type PrP (PrP^Wt^), suggesting pathological PrP^Q227X^ is incapable of recruiting PrP^Wt^ in vivo. This mutant anchorless protein, however, is able to recruit PrP^Wt^ from humanized transgenic mouse brain but not from autopsied human brain homogenates to produce a protease-resistant PrP^Sc^-like form in vitro by protein misfolding cyclic amplification (PMCA). To further investigate the characteristics of this mutation, constructs expressing human PrP^Q227X^ or PrP^Wt^ were transfected into neuroblastoma cells (M17). Fractionation of the M17 cells demonstrated that most PrP^Wt^ is recovered in the cell lysate fraction, while most of the mutant PrP^Q227X^ is recovered in the medium fraction, consistent with the results obtained by immunofluorescence microscopy. Two-dimensional gel-electrophoresis and Western blotting showed that cellular PrP^Q227X^ spots clustered at molecular weights of 22–25 kDa with an isoelectric point (*p*I) of 3.5–5.5, whereas protein spots from the medium are at 18–26 kDa with a *p*I of 7–10. Our findings suggest that the role of GPI anchor in prion propagation between the anchorless mutant PrP and wild-type PrP relies on the cellular distribution of the protein.

## Introduction

The cellular prion protein (PrP^C^) is a glycoprotein that is attached to the cell surface by a glycosylphosphatidylinositol (GPI) anchor. Although its physiological function remains unclear, the prion protein is notoriously associated with prion diseases, a group of transmissible spongiform encephalopathies (TSEs) affecting both animals and humans, which include Creutzfeldt-Jakob disease (CJD), Gerstmann-Sträussler-Scheinker (GSS) syndrome, fatal insomnia, and variably protease-sensitive prionopathy (VPSPr) in humans, as well as scrapie in sheep and goats, bovine spongiform encephalopathy (BSE) or mad cow disease in cattle, and chronic wasting disease in deer and elk (see [1] for review). These diseases are all associated with the key molecular event of the template-assisted conversion of PrP^C^ into its pathological isoform PrP^Sc^ [2], which is believed to take place mainly on the cell surface [3–5], mostly likely involving the PrP GPI anchor to make both PrP^C^ and PrP^Sc^ interacting with each other physically and functionally.

Previous studies investigating the role of the GPI anchor in PrP^Sc^ propagation, however, have generated controversial results. Cell-free experiments have revealed that anchorless PrP can be efficiently converted into a proteinase K (PK)-resistant PrP^Sc^-like form (PrP^res^) [6, 7]. Moreover, it has been shown that in mouse neuroblastoma cells (ScN2a) expression of C-terminally truncated PrP without the GPI anchor did not inhibit formation of anchorless PrP^Sc^ [8]. On the other hand, most studies with cell models have shown that the GPI anchor is required for PrP^Sc^ formation. For example, cells expressing anchorless PrP^C^ were shown to be unable to support persistent scrapie infection [4], and treatment with phosphoinositide-specific phospholipase (Pl-PLC), which cleaved the GPI anchor, diminished PrP^Sc^ formation, and cleared prion infection in ScN2a cells [3, 9]. Furthermore, treatment with glimepiride, a sulfonylureal antidiabetic agent previously shown to upregulate endogenous PI-PLC, was found to significantly decrease the level of PrP^Sc^ in three infected neuronal cell lines [10, 11].

Interesting studies by Chesebro and co-workers with transgenic (Tg) mice expressing GPI-anchorless PrP revealed that absence of the GPI anchor differentially influenced development of prion disease and prion infectivity [12–14]. Specifically, GPI-anchorless PrP Tg mice were found to be susceptible to infection by three scrapie strains, including RML, ME7, and 22L, confirmed by the presence of detectable PrP^res^ and PrP amyloid plaques in the brain by Western blotting and histology; but these mice developed virtually no clinical signs of prion disease during their lifespan [12]. In contrast, co-expression of wild-type PrP (PrP^Wt^) with GPI-anchorless PrP resulted in accelerated progression of scrapie disease after prion inoculation. Their subsequent study with homozygous Tg mice expressing two-fold more anchorless PrP revealed that scrapie infection induced a new fatal disease with unique clinical signs and altered neuropathology in these Tg mice compared with wild-type control mice [13]. A combination of these findings clearly indicated that the presence of the anchored PrP^C^ on the cell surface is a prerequisite for the pathogenesis of prion disease. In a particularly interesting case, a patient with GSS was reported to be associated with heterozygous PrP nonsense mutation of Q227X in which the patient carried both anchorless mutant PrP and PrP^Wt^ alleles [15]. As in the infected anchorless PrP-expressing Tg mice, the presence of numerous amyloid plaques without significant spongiform degeneration was observed in the patient-characteristic of GSS, especially with Western blot detection of a 7 kDa PrP^res^. However, in contrast to typical GSS, the patient exhibited a clinical phenotype more resembling frontotemporal dementia, without ataxia or pyramidal signs, and with relative sparing of the cerebellum [15].

To expand our understanding of the role of the GPI anchor in PrP^Sc^ formation and PrP trafficking, we characterized the biochemical features of PrP^Sc^ from this unique case and human anchorless PrP expressed in human neuroblastoma cell lines.

## Materials and Methods

### Ethics Approval and Consent to Participate

The use of autopsy human brain tissues was authorized by the Institutional Review Board of University Hospital Cleveland Medical Center and the Case Western Reserve University, Cleveland, Ohio, USA, or by the Medical Ethics Committee of the University Medical Centre Utrecht 11-531/C, the Netherlands. The consents were received for each case through the Dutch Surveillance Centre for Prion Diseases (DSCPD), Utrecht, Netherlands, or the National Prion Disease Pathology Surveillance Center (NPDPSC), Case Western Reserve University, Cleveland, Ohio, USA, for research on retained tissues after written informed consents given by the patients during life or their next of kin after death. Postmortem examinations, if permission was available, were carried out in DSCPS or NPDPSC. All information was analyzed anonymously. Animal experiments in this study were approved by the Institutional Animal Use and Care Committee and the Institutional Biosafety Committee at the Case Western Reserve University.

### Reagents and Antibodies

Proteinase K (PK), 1-hydroxybenzotriazole, N, N′-diisopropylcarbodiimide, trifluoroacetic acid, triisobutylsilane, piperidine, and dichloromethane were obtained from Sigma Chemical Co. (St. Louis, MO). Peptide N-glycosidase F (PNGase F) and protease inhibitor cocktail were obtained from New England Biolabs (Beverly, MA). Fmoc-amino acids and amino-PEG cellulose membranes were obtained from Intavis (San Marcos, CA). N-α-Fmoc-O-benzyl-L-phosphothreonine and N-α-Fmoc-O-benzyl-L-phosphoserine were purchased from AnaSpec (San Jose, CA). ECL Plus for enhanced chemiluminescence was purchased from Amersham Pharmacia Biotech, Inc. (Piscataway, NJ). The following anti-PrP antibodies were used: 3F4 directed against human PrP residues 106–110 [16], 1E4 against human PrP97–105 [17, 18] (Cell Sciences, Inc., Canton, MA), and anti-C against PrP220–231 [17, 18]. Anti β-tubulin antibody was purchased from Abcam (Cambridge, MA).

### Cloning and Production of Cell Lines and Preparation of Cell Lysate

M17 cells, the SK-N-Be(2) neuroblastoma cell line isolated from a 2-year-old male (ATCC, Manassas, VA), were transfected with an episomal vector, CEP4β, containing human prion protein-coding sequences of PrP^Q227X^, PrP^Wt^, or PrP^T183A^ that is linked to a familial CJD [19] using the cationic lipid DOTAP (Roche Applied Science), as previously described [20, 21]. Transfected cells were cultured as bulk selected hygromycin-resistant cultures at 37 °C in OPTI-MEM with 5% calf serum supplement and iron-enriched (GIBCO-BRL) and 500 μg/mL Hygromycin B (Calbiochem, La Jolla, CA). Cells were trypsinized from the flask and counted. The same number of cells were seeded onto Petri plates for each cell culture. Cells were incubated to ~ 95% confluence in complete medium supplemented with fetal bovine serum (FBS) (Thermo Fisher Scientific Inc., Waltham, MA) and antibiotics. For experiments in which culture media was collected for detection of secreted PrP, no FBS was added to the culture media. To prepare cell lysates, cells were rinsed with PBS three times and lysed in 1.0 mL of lysis buffer (10 mM Tris, 150 mM NaCl, 0.5% Nonidet P-40, 0.5% deoxycholate, 5 mM EDTA, pH 7.4) on ice for 30 min after removing the media. Nuclei and cellular debris were removed from cell lysates by centrifugation at 1000 ×*g* for 10 min at 4 °C. The supernatant was incubated with 5.5 mL of pre-chilled methanol at − 80 °C for 2 h and centrifuged at 14,000 ×*g* for 30 min at 4 °C. The pellet was resuspended in 100 μL of lysis buffer for future analysis.

### Brain Tissues and Preparation of Brain Homogenates

As previously described [15], the patient was a 42-year-old woman and autopsy was performed after informed consent was signed and the explicit permission to use tissues for research was obtained. The brain tissue was collected approximately 8 h after death. Other frozen brain tissues were collected from sporadic CJD cadavers provided by our NPDPSC, Case Western Reserve University, Cleveland, Ohio, USA. The 10% (*w*/*v*) brain homogenates from different brain areas were prepared in 9 volumes of lysis buffer (10 mM Tris, 100 mM NaCl, 0.5% Nonidet P-40, 0.5% deoxycholate, 10 mM EDTA, pH 7.4). Brain homogenates were centrifuged at 1000 ×*g* for 10 min at 4 °C, and the supernatant was collected (S1). For PK treatment, tissue homogenates were incubated with designated amounts of PK at 37 °C for 1 h and the digestion was terminated by adding protease inhibitor cocktail (Millipore Sigma, St. Louis, MO, UA) at a final concentration of 1× solution of 1:100 dilutions and boiling in SDS sample buffer (3% SDS, 2 mM EDTA, 4% β-mercaptoethanol, 10% glycerol, 50 mM Tris, pH 6.8) for 10 min.

### Protein Misfolding Cyclic Amplification

The preparation of PrP seeds and substrates, as well as protein misfolding cyclic amplification (PMCA) assay, were conducted as previously described [22–24]. Briefly, brain tissues of humanized Tg mice expressing human PrP with 129 polymorphism methionine (M)/M (Tg40h) or PrP-valine (V)/V (TgWV), or autopsied normal human brain tissues from cadavers carrying PrP-12MM or 129VV were carefully dissected to avoid blood contamination as much as possible [22, 23]. Cell lysates from M17 cells expressing human wild-type PrP-129M were also used as substrates. To prepare the frozen normal Tg mouse and human brain homogenate for use as the substrate, brain tissues were homogenized (10% *w*/*v*) in PMCA conversion buffer [150 mM NaCl, 1% Triton X-100, 8 mM EDTA, pH 7.4, and the complete protease inhibitor mixture cocktail (Roche) in PBS]. The brain tissue homogenates from GSS^Q227X^ and sCJD containing PrP^Sc^ seeds were prepared as described previously for the brain substrates. Tissue homogenates were centrifuged at 500 ×*g* for 10 min at 4 °C, and the supernatant (S1) fraction was collected for use as the substrate or centrifuged at 500 ×*g* for 3 min for the brain samples used as seeds. The samples were kept at − 80 °C until use. Each seed was diluted with the substrate at a 1:100 ratio (1 μL seed + 99 μL substrate) in 200 μL PCR tubes with a PTFE bead (diameter 3/32 in.) (Teflon, APT, RI). A total of 20 μL of each mixture was removed and kept at − 20 °C as a non-PMCA control. The remainder of the samples was subjected to PMCA. Each PMCA cycle comprised a 20 s elapsed time of sonication at amplitude 85 (250 watts; Misonix S3000 sonicator) followed by an incubation period of 29 min and 40 s at 37 °C as previously described [22, 23]. After PMCA, the samples were subjected to Western blotting for detection of PrP^res^ as described subsequently.

### Velocity Sedimentation in Sucrose Step Gradients

Brain homogenates or cell lysates were incubated with an equal volume of 2% Sarkosyl for 30 min on ice. The samples were loaded on 10–60% sucrose step gradients and centrifuged at 200,000 ×*g* in a SW55 rotor for 1 h at 4 °C as described with minor modification [17, 21, 25]. After centrifugation, the samples were sequentially removed from the top to the bottom of the centrifuge tubes to collect 12 equal fractions. Aliquots of the 12 fractions were subjected to immunoblot analysis as described subsequently to determine the amount of PrP in each fraction.

### Immunofluorescence Microscopy

Immunofluorescence staining of PrP was conducted as previously described [21] with minor modification. Briefly, transfected cells expressing PrP^Q227X^, PrP^Wt^, or PrP^T183A^ were grown overnight on poly-D-lysine coated coverslips. The cells were fixed in 4% paraformaldehyde for 15 min at room temperature. Non-specific binding was prevented by incubating slides with PBS-T [10% goat serum (Thermo Fisher Scientific Inc., Waltham, MA), 2% T-20, 1% Triton X-100], for 1 h at room temperature before rinsing with PBS. The slides were then incubated with 1E4 (1:50) at room temperature, rinsed with PBS, and followed with FITC-conjugated goat anti-mouse IgG at 1:320 (Sigma, St. Louis, MO) for 1 h at room temperature in the dark. Following washing with PBS, the coverslips were mounted on the immunostained slides using fluorescent mounting medium fluoromount with DAPI (Dako, Carpinteria, CA).

### Western Blot Analysis

Samples treated for Western blotting were resolved on 15% Tris–HCl Criterion pre-cast gels (Bio-Rad) for SDS polyacrylamide gel electrophoresis at 150 V for ~ 90 min. The proteins in the gels were transferred to Immobilon-P membrane (PVDF, Millipore) for ~ 90 min at 70 V. The membranes were incubated for 2 h at room temperature with either 3F4 (1:40,000), anti-C (1:10,000), or anti-β tubulin (1:3000) as the primary antibody for probing the PrP molecule. Following incubation with HRP-conjugated sheep anti-mouse IgG at 1:5000 for the monoclonal antibody 3F4 or donkey-anti-rabbit IgG for anti-C or anti-β tubulin at 1:6000, the protein bands or spots were visualized on Kodak film using ECL Plus according to the manufacturer’s protocol.

### Statistical Analysis

The statistical differences in intensity of PrP among different groups detected by Western blotting or immunofluorescence microscopy were statistically analyzed using Student’s *t* test to obtain *p* values. All tests adopted a two-sided type I error level of 0.05.

## Results

### PK-Resistant PrP^Sc^ From the Brain of the Patient With PrP^Q227X^ Is Detectable by 3F4 But Not by Anti-C Antibody With Western Blotting

**Fig. 1:**
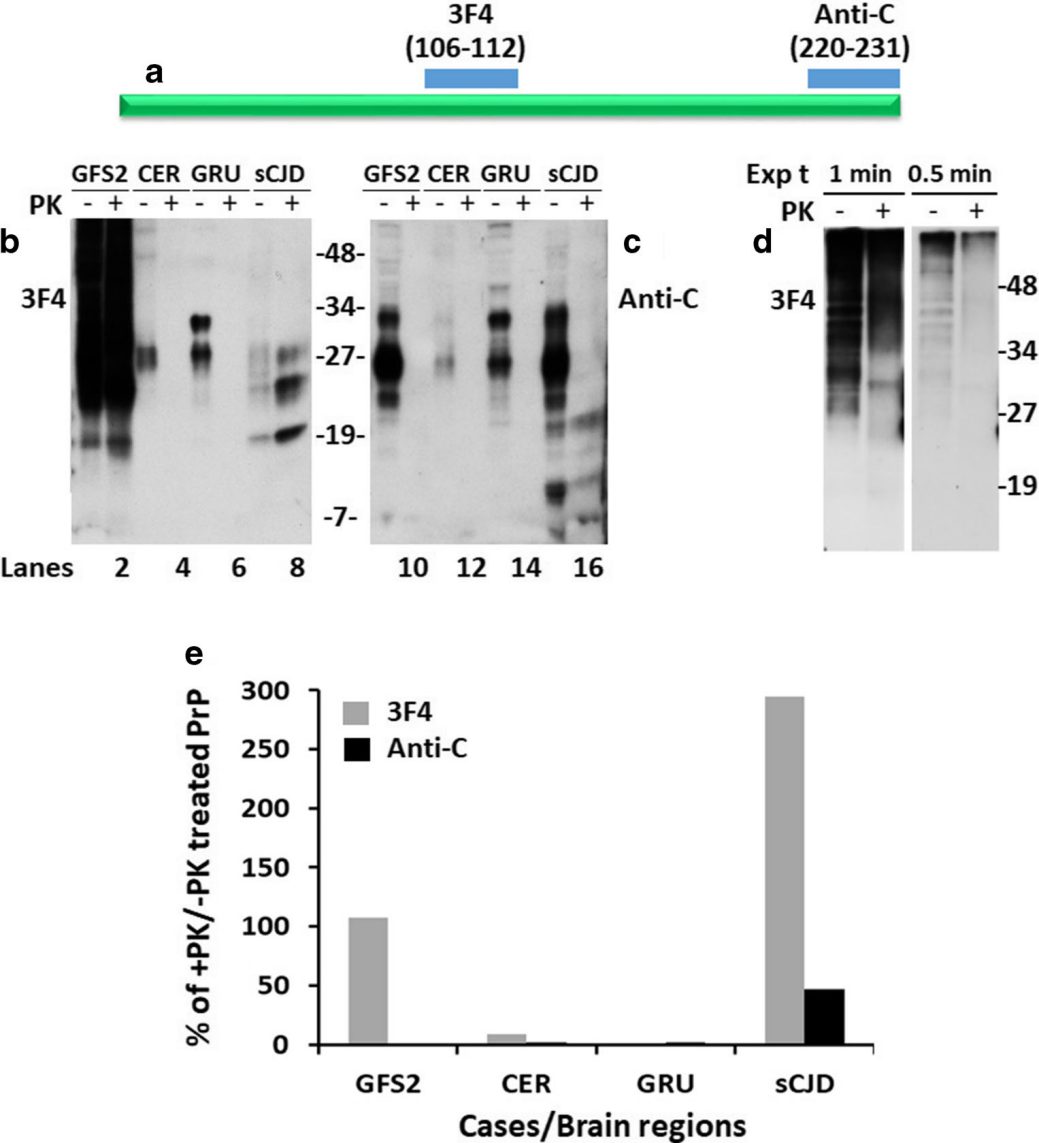
PK-resistant PrP^Sc^ from the brain of the patient with PrP^Q227X^ is detectable by 3F4 but not by anti-C antibody with Western blotting. (**a**) Schematic diagram showing the epitopes of 3F4 and anti-C antibodies localized at PrP106–112 and PrP220–231, respectively. Three brain areas from the patient with PrP^Q227X^: GFS2, frontal superior gyrus; CER, cerebellar hemisphere; GRU, gyrus rectus. PrP^Sc^ from three different brain areas of the patient with PrP^Q227X^ mutation were treated with or without PK prior to Western blot probing with antibodies 3F4 (**b**) and anti-C (**c**). (**d**) Short exposure time (Exp t) of Western blot of GFS2 probing with 3F4. (**e**) Bar graph showing the percentage of PK treated to non-treated PrP in each sample quantitated by densitometric analysis

According to the previous study [15], the PrP^Sc^ molecule from the patient with PrP^Q227X^ is biochemically similar to that of GSS in that it produces no clear PrP^Sc^ type 1 or type 2 bands, but rather smeared PrP bands detectable from the top of the gel down to a molecular weight of 7 kDa. To further characterize the PrP^Sc^ molecule, we first analyzed the protein from three different brain regions of this patient, the frontal superior gyrus (GFS2), cerebellar hemisphere (CER), and the gyrus rectus (GRU), by Western blotting. Brain homogenates were treated with or without PK digestion prior to Western blot analysis probing with the widely used anti-PrP monoclonal antibody 3F4 directed against PrP residues 106–112 [16] (Fig. 1a). The highest amount of PrP from non-PK treated samples was detected in the GFS2 brain sample among three different brain regions analyzed (Fig. 1b). In contrast to the PrP gel profile from the sCJD control, but similar to the previous observation [15], a smear of high molecular PrP bands starting at the top gel was detected, which may represent aggregated PrP molecules. Moreover, the smear PrP gel profile was not changed by PK treatment (Fig. 1b). In contrast, no PrP^res^ was detected after PK digestion in the samples from CER and GRU. To determine whether there are the classic triad of PrP27–30 fragments with variable glycosylation commonly seen in sCJD, we decreased the exposure time of films to 1 and 0.5 min, which confirmed no classic triad of PrP bands instead of multiple PrP bands from the top of the blots in this peculiar case (Fig. 1d).

PrP^Sc^ has been reported to be composed of both wild-type and mutant proteins or only mutant proteins in familial prion diseases, including GSS [26–34]. To determine whether PrP^Sc^ in GSS with this unique anchorless-causing mutation derives from both wild-type and mutant PrP alleles or from mutant allele alone, we examined the PrP^Sc^ molecule using an antibody directed against the C-terminal domain 220–231 of PrP, anti-C antibody [17], whose epitope includes PrP residue 227, the site of the PrP Q227X mutation (Fig. 1a). When the blot probed with the 3F4 antibody described previously was reprobed with the anti-C antibody, PrP was detected in all samples but PrP^res^ was only observed in the brain homogenate from sCJD; no PrP^res^ was detected in the sample from GFS2 of this case (Fig. 1c). It is worth noting that while both PK-treated and untreated PrP from GFS2 were detectable by 3F4, only PK-untreated PrP was detected by anti-C. It is expected that the samples before PK treatment were composed of both PrP^C^ and PrP^Sc^ while the samples after PK treatment mainly contained PrP^Sc^. Given that the mutation of Q to X at PrP residue 227 falls exactly within the epitope of the anti-C antibody between residues 220 and 231 of PrP, but not the epitope of the 3F4 antibody (PrP106–112) (Fig. 1a), it is conceivable that the 3F4 antibody detected both wild-type and mutant PrP while the anti-C antibody detected only PrP^Wt^, but not mutant PrP (PrP^Mut^). Therefore, the results indicate that PrP^Sc^ from GSS linked to the PrP^Q227X^ mutation is most likely composed of mutant protein, similar to other forms of GSS [26, 30, 32].

### PrP Aggregates Isolated by Sucrose Gradient Sedimentation From the Brain of the Patient With PrP^Q227X^ Are Mainly Detectable by 3F4 But Not by Anti-C

**Fig. 2:**
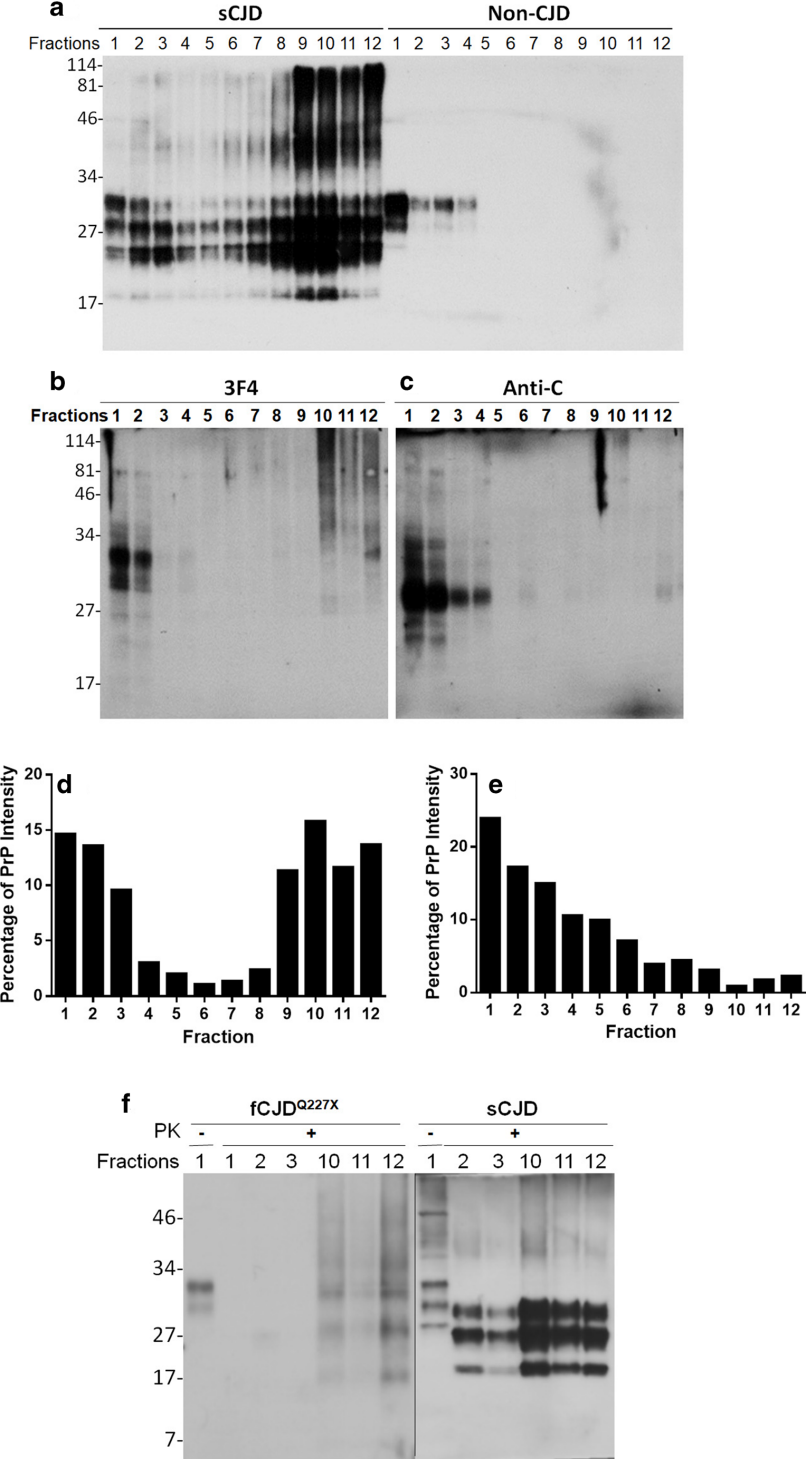
PrP aggregates isolated from brain homogenate of the patient with GSS^Q227X^ by sucrose gradient sedimentation are detectable by 3F4 but virtually not by anti-C. Representative Western blotting of PrP in different fractions of sucrose gradient sedimentation from brain homogenates of subjects with sCJD (**a**, left side), non-CJD control (**a**, right side), or GSS^Q227X^ (**b** and **c**, GFS2). Bar graphs showing the ratio of PrP intensity in each sucrose gradient sedimentation fraction to the total PrP intensity in all fractions from Western blot detected by 3F4 (**d**) or anti-C (**e**). (**f**) Representative Western blotting of PK-treated PrP from sucrose gradient top fractions (1, 2, and 3) and bottom fractions (10, 11, and 12) of brain homogenates of GSS^Q227X^ (left panel) and sCJD (right panel). Panels **a**, **b**, and **f** were probed with 3F4, while panel **c** was probed with anti-C. Molecular markers are indicated on the left side of the blots

Sucrose gradient sedimentation analysis has been widely used to separate PrP^Sc^ aggregates from their monomers based on their size, density, and shape [17, 21, 25]. While the detergent-soluble and monomeric PrP^C^ from non-CJD brain homogenate was only detectable in the top fractions 1–4 (Fig. 2a, right side), the detergent-insoluble PrP^Sc^ aggregates from sCJD brain homogenate were mostly recovered in the bottom fractions 8–12 (Fig. 2a, left side) after sucrose gradient sedimentation. Notably, Western blotting showed that the PrP molecules in the bottom fractions 8–12 contained not only PrP bands migrating between 34 and 17 kDa representing the PrP^Sc^ monomers from the dissociated PrP aggregates, but also PrP bands migrating between 114 and 34 kDa representing the PrP^Sc^ oligomers that are resistant to the SDS-induced dissociation. To determine whether the PrP^Sc^ aggregates contain either both PrP^Q227X^ and PrP^Wt^ or PrP^Q227X^ alone, the brain homogenate of the patient with PrP^Q227X^ was also subjected to sucrose gradient sedimentation assay followed by Western blotting of PrP in sedimentation fractions probed with the 3F4 and anti-C antibodies. On the 3F4 blot, the PrP molecules were observed in both top and bottom fractions (Fig. 2b, d) as those in sCJD (Fig. 2a). However, in contrast to sCJD, PrP bands in the bottom fractions from GSS^Q227X^ were mostly detected between 114 and 34 kDa but not between 34 and 17 kDa by 3F4 (Fig. 2b). Moreover, when the blot was re-probed with the anti-C antibody, PrP was predominantly detected in the top fractions 1–4 and only faint bands were found in the bottom fractions (Fig. 2c, e). The finding that the PrP recovered in the bottom fractions was detected only by 3F4 but virtually not by anti-C suggests that these PrP aggregates are composed of the mutant PrP^Q227X^ molecule since anti-C exhibited a poor affinity to PrP^Q227X^ as indicated previously. Densitometric analysis of PrP intensity of each fraction revealed that more than 95% PrP detected by anti-C was recovered in the top fractions, suggesting PrP^Sc^ in GSS^Q227X^ is mostly derived from PrP^Q227X^ (Fig. 2e). These observations are in good agreement with the finding shown in Fig. 1. After the PK treatment, PrP^res^ from GSS^Q227X^ was virtually detectable only in the bottom fractions 10–12 but not in top fractions 1–3; moreover, the gel profile of the PK-resistant PrP bands in the bottom fractions of GSS^Q227X^ brain homogenates was significantly different from that of sCJD (Fig. 2f). The former exhibited atypical PrP bands from the top to the bottom of the gel while the latter was found to have the typical PrP27–30 three bands migrating at between 30 and 17 kDa.

### PrP^Sc^ From GSS^Q227X^ Is Able to Recruit PrP From Humanized Tg Mouse Brain But Not PrP From Autopsy Human Brain Homogenates by Protein Misfolding Cyclic Amplification

**Fig. 3:**
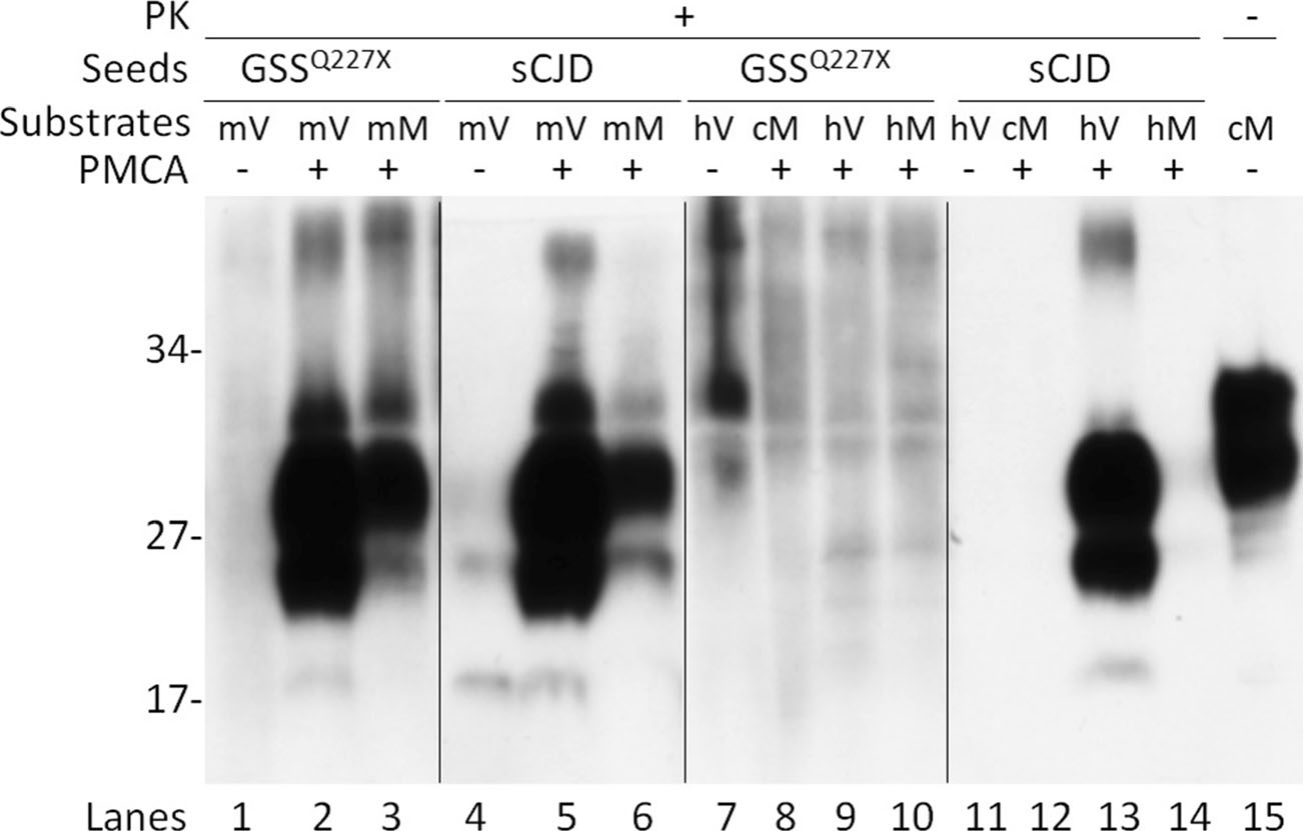
PrP^Sc^ from GSS^Q227X^ converts wild-type human PrP from humanized Tg mouse brains but not PrP from normal human brains in vitro by PMCA. Western blot analysis of PK-resistant PrP amplified by PMCA with brain homogenates as seeds from GSS^Q227X^ (lanes 1–3 and 7–10) or sCJD (lanes 4–6 and 11–14) with different PrP^C^ substrates. These substrates include either normal brain homogenates from humanized TgWV expressing human wild-type PrP-129VV polymorphism (mV, lanes 2 and 5), Tg40h expressing human PrP-129MM (mM, lanes 3 and 6), normal human brain homogenates containing PrP-129VV polymorphism (hV, lanes 9 and 13) or PrP-129MM polymorphism (hM, lanes 10 and 14), or cell lysates from M17 cell expressing human wild-type PrP-129MM (cM, lanes 8 and 12). Molecular weights are indicated on the left side of the blots. Cell lysates (cM) without PK treatment and PMCA were used as a control in lane 15. Probed with 3F4

Next, we determined whether PrP^Sc^ from GSS linked to PrP^Q227X^ mutation is able to convert wild-type human PrP with either 129VV (PrP-129V) or 129MM (PrP-129M) polymorphism in vitro by protein misfolding cyclic amplification (PMCA). The wild-type human PrP^C^ molecule used as a substrate in PMCA was from brain homogenates of humanized Tg mice expressing wild-type human PrP carrying 129VV (TgWV, mV) or PrP-129MM (Tg40h, mM), autopsy human brain homogenates carrying PrP-129VV (hV) or PrP-129MM (hM), or M17 cell lysates transfected with human PrP-129MM (cM) as described previously [22, 23]. In the presence of PrP^C^ from brain homogenates of humanized Tg mice (mV or mM) as the substrate, PMCA produced intense PrP^res^ in the PMCA samples (Fig. 3, lanes 2, 3, 5, and 6) compared with the non–PMCA-treated samples (Fig. 3, lanes 1 and 4), indicating PrP^Sc^ from GSS^Q227X^ was able to convert humanized Tg mouse-derived wild-type human PrP-129VV (Fig. 3, lanes 2 and 5) and PrP-129MM (Fig. 3, lanes 3 and 6) in vitro by PMCA. More PK-resistant PrP was generated with mV than with mM as a substrate (Fig. 3, lanes 2 and 5 vs lanes 3 and 6). The gel profile of PK-resistant PrP amplified from GSS^Q227X^ was similar to that of PrP amplified from a sCJDVV2 control (Fig. 3, lanes 2 and 3 vs lanes 5 and 6), showing predominant upper and middle bands and underrepresented low band. In contrast, compared with non-PMCA control (Fig. 3, lane 7), no PrP^res^ was amplified by PMCA from GSS^Q227X^ seeds in the presence of PrP^C^ from autopsied normal human brain homogenates (hV or hM) or cell lysates (cM) as a substrate (Fig. 3, lanes 7–10). PrP^Sc^ from the sCJDVV2 control was amplified only in the hV substrate (Fig. 3, lane 13), but not in cM and hM substrates (Fig. 3, lanes 12 and 14). Taken together, the PMCA results indicate that PrP^Sc^ from GSS^Q227X^ was unable to convert wild-type PrP^C^ from normal human brain homogenates or cultured cells while it converted wild-type PrP^C^ from humanized Tg mouse brain homogenates in vitro.

### More PrP^Q227X^ Is Secreted into Cell Culture Medium Than Wild-Type PrP

**Fig. 4:**
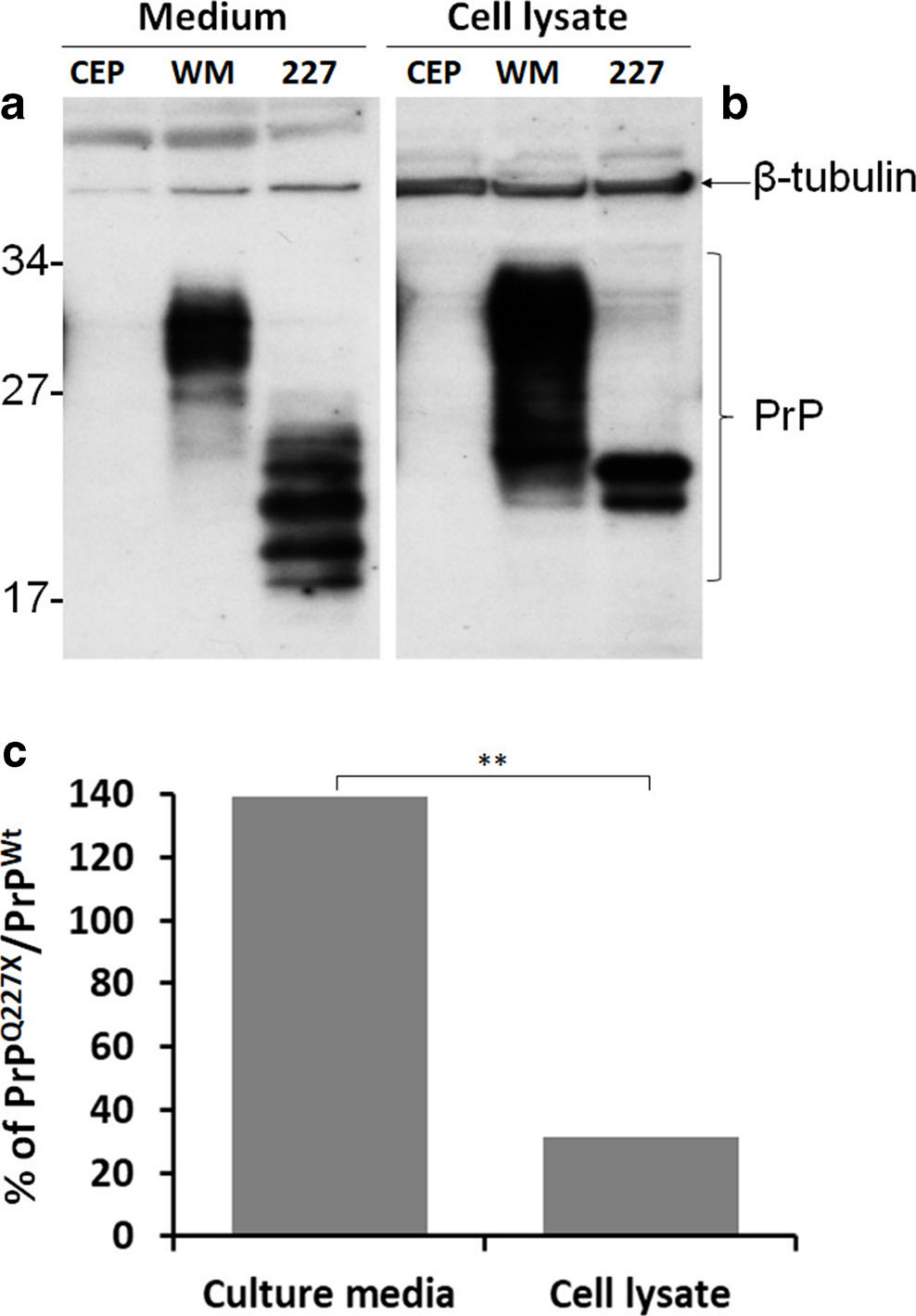
PrP^Q227X^ is less glycosylated and mainly released to culture medium. PrP from cell culture media (**a**) or cell lysates (**b**) of cultured M17 human neuroblastoma cells transfected with no human PrP (CEP), or with wild-type human PrP-129M (WM), or human PrP^Q227X^ mutation (227) was examined by Western blot probing with the 3F4 antibody. The loading amount in each sample was monitored using β-tubulin as an internal loading control. (**c**) Quantitative analysis of PrP in culture medium or cell lysate from cells transfected with human PrP^WM^ or PrP^Q227X^ by densitometric analysis. **p < 0.01

To explore the biochemical properties of PrP with Q227X mutation, human PrP^Q227X^ was transfected into the human neuroblastoma cell line M17. M17 cells expressing wild-type human PrP carrying the 129M polymorphism (WM, or PrP^WM^) or transfected with empty vector (CEP) were used as controls. PrP collected from cell culture media and cell lysates was examined by Western blotting. As expected, the migration of PrP^Wt^ from media and cell lysates was between molecular weights of approximately 34 and 19 kDa (Fig. 4a, b). In contrast, migration of PrP^Q227X^ bands from the culture media was between 27 and 15 kDa as well as between 24 and 21 kDa from cell lysates (Fig. 4a, b). There were 6–7 PrP bands in the culture media and 2 bands in the cell lysates. The ratio of PrP^Q227X^ to PrP^WM^ was significantly higher in the culture media than in cell lysates (Fig. 4c), suggesting that more PrP^Q227X^ was secreted into cell culture media, probably due to the lack of PrP GPI anchor as a result of the Q227X stop mutation. Moreover, the molecular weight of PrP^Q227X^ was significantly smaller than that of PrP^WM^, suggesting that predominant glycoform of PrP^Q227X^ is unglycosylated, which is in agreement with the previous report that anchorless PrP expressed in transgenic mice was less glycosylated [12]. Moreover, our finding that more anchorless PrP was released into medium is consistent with the observation by Campana et al. [35] that more than 90% of anchorless PrP^C^ was either released into the culture medium or accumulated in the Golgi apparatus.

### The PrP^Q227X^ Molecules Released Into Culture Medium and Remaining in Cells Exhibit Different Two-Dimensional Gel Electrophoresis Profiles

**Fig. 5:**
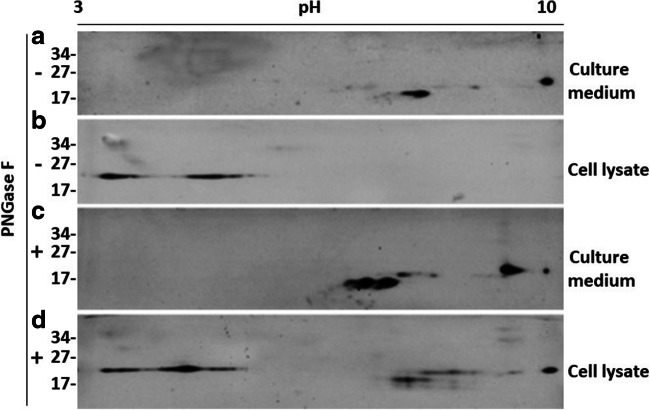
The PrP^Q227X^ molecules released into culture medium and associated with cells exhibit different 2D gel profiles. The upper two panels **a**, **b** show the two-dimensional (2D) Western blotting (WB) of PrP from cell culture media or cell lysates of cultured M17 cells transfected with human PrP^Q227X^ without PNGase F treatment. The lower two panels **c**, **d** display the 2D blots of PrP^Q227X^ that was treated with PNGase F prior to WB. The molecular weights are indicated on the left side of blots

To compare the PrP^Q227X^ molecule from culture medium and cell lysate of M17 cells, samples were analyzed using two-dimensional (2D) gel electrophoresis and Western blotting, an assay that separates proteins based on both molecular charge and weight. On the 2D blots, the medium-derived PrP^Q227X^ exhibited two intense spots and a single weak spot migrating at 18–26 kDa with isoelectronic points (*p*I) from 7 to 10 (Fig. 5a). In contrast, the PrP^Q227X^ molecule isolated from cell lysates showed smeared spots migrating at virtually the same molecular weight of 22–25 kDa with *p*I from 3.5 to 5.5 (Fig. 5b). After deglycosylation with peptide-N-glycosidase F (PNGase F), the molecular weight of PrP from culture medium decreased slightly from 18–26 kDa to 15–25 kDa and the *p*I shifted toward between 6.5 and 9.5 (Fig. 5c). However, while the lysate PrP^Q227X^ spots remained in the similar positions as shown in Fig. 5b migrating at 22–25 kDa with pI 3.5–5.5, additional deglycosylated PrP spot clusters were observed migrating at 17–25 kDa with *p*I similar to those detected in the culture medium as shown in Fig. 5a, c and d). These results suggested that there were two groups of PrP^Q227X^ molecules: one clustered at molecular weights of 18–26 kDa with *p*I 7–10 and the other clustered at molecular weight of 22–24 kDa with *p*I 3.5–5.5. The former was mainly released into cell culture medium while the latter remained within the cells. It is unclear why PrP^Q227X^ from cell lysate did not show PrP spots with *p*I 7–10 in the samples without PNGase F treatment. Whether the new spots resulted from the exposure of the epitope after deglycosylation or from less glycosylated isoforms remains to be determined.

### The Amount of PrP on the Cell Surface Is Significantly Lower in Cells Expressing PrP^Q227X^ Than in Cells Expressing PrP^Wt^

To explore the cellular distribution of PrP^Q227X^ in M17 neuroblastoma cells, we compared the cells expressing human PrP^Q227X^ (227) with cells expressing normal wild-type PrP (PrP^Wt^, WM) or mutant PrP^T183A^ (183), a mutation linked to familial CJD [19]. We have previously shown that PrP^Wt^ is predominantly attached to the cell surface, whereas PrP^T183A^ accumulates intracellularly and was unable to reach the cell surface [21, 36]. These cells were analyzed by immunofluorescence microscopy with the anti-PrP antibody 1E4 in the absence of Triton-X 100 in order to stain only the PrP on the cell surface since the antibody is unable to cross cell membrane to enter the cytosol without Trion-X 100 treatment [21, 36].

**Fig. 6:**
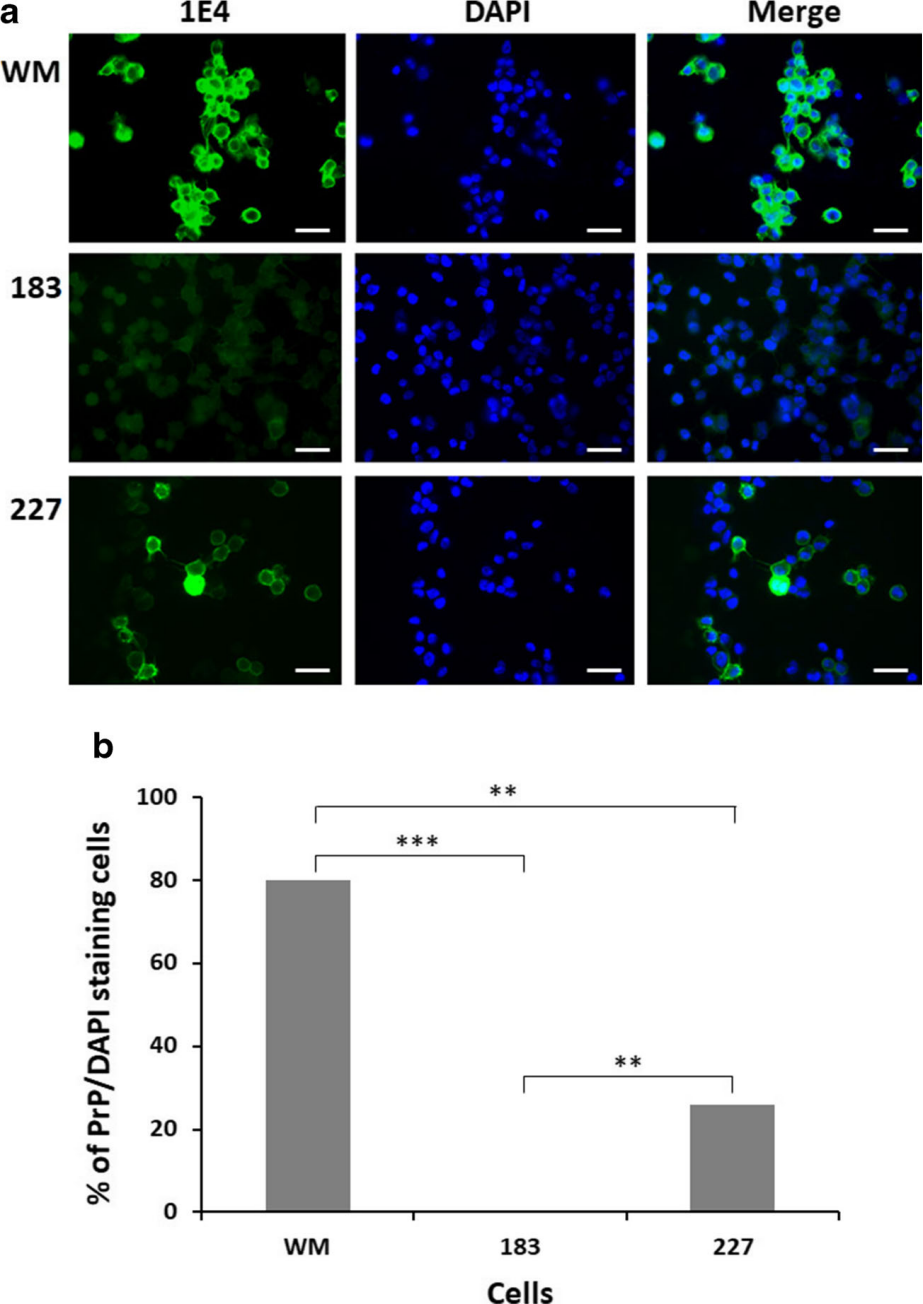
The amount of PrP on the cell surface is significantly lower in cells expressing PrP^Q227X^ than in cells expressing PrP^Wt^. (**a**) Neuroblastoma cells that were mock transfected (CEP) or transfected with either PrP^WM^ (WM), PrP^T183A^ (183), or PrP^Q227X^ (227) were subjected to immunofluorescence microscopy with the anti-PrP antibody 1E4 in the absence of Triton-X 100 to stain only PrP on the cell surface (left panels). The middle panels show DNA staining with DAPI, and the panels on the right are the merged images of the left and middle panels. (**b**) Quantitative analysis of ratio of cells stained with PrP/stained with DAPI. **p < 0.01; ***p < 0.001

More than 90% cells showed PrP staining on the cell surface in M17 cells expressing PrP^Wt^ while virtually no PrP staining was found in cells expressing PrP^T183A^ (Fig. 6a, top and middle panels, Fig. 6b). However, less than 30% of cells expressing PrP^Q227X^ exhibited PrP staining (Fig. 6a, low panels, Fig. 6b), suggesting that although most of the anchorless PrP^Q227X^ molecules were released into cell culture medium, it still could be found associated with the cell surface in a small population of cells.

## Discussion

Several lines of investigation using cell-free, cell, and animal models provided evidence that the PrP GPI anchor plays important roles in the pathogenesis of prion disease and/or PrP^Sc^ formation by making PrP^C^ available for PrP^Sc^ propagation on the cell surface leading to neuronal cell dysfunction/death. However, there were a few conflicting findings and there was a lack of studies examining anchorless PrP in humans. In this study, with brain tissues of a previously reported GSS patient carrying a PrP stop mutation, Q227X, and human neuroblastoma cells expressing human PrP^Q227X^ or PrP^Wt^, we made the following observations: First, PrP^Sc^ in GSS^Q227X^ is mainly composed of mutant protein in large aggregates. Second, PrP^Sc^ in GSS^Q227X^ is unable to act as a seed for converting PrP^Wt^ from normal human brains while it converts wild-type PrP^C^ from humanized Tg mouse brain homogenates. Third, most anchorless PrP^Q227X^ is released into cell culture medium while a small percent remains associated with the cells. Finally, the PrP^Q227X^ molecules isolated from cells and in the culture medium showed significant differences in *p*I: cells at 3.5–5.5 and culture medium at 7–10. This study provides insight on the effect of the GPI anchor on the synthesis and trafficking of human PrP in a human prion disease, GSS^Q227X^, and in cultured cell models, improving our understanding of the role of the GPI anchor in the pathogenesis of human prion disease.

PrP^Sc^ has been found to be derived from both mutant and wild-type PrP molecules or from mutant protein alone in inherited human prion diseases [26–34]. While PrP^Sc^ in most types of genetic CJD is composed of both mutant and wild-type PrP molecules, PrP^Sc^ associated with most types of GSS consists of mutant protein alone. These interesting preferences may suggest that the ability of PrP^Sc^ to recruit its normal counterpart PrP^Wt^ is dependent on the specific mutations. Our antibody mapping indicated for the first time that the PK-resistant PrP^Sc^ found in the GSS^Q227X^ patient follows the preference and mainly contained mutant protein, implying PrP^Q227X^ does not seed or seeds less efficient conversion of PrP^Wt^ into PrP^Sc^. Since the Q227X mutation is expected to abolish the PrP GPI anchor, the mutant protein is unable to attach to the cell surface. As a consequence, the mutant protein is not physically in contact with the PrP^Wt^ molecule. Moreover, our sucrose gradient study revealed that while PrP^Wt^ is soluble and monomeric and recovered in the top fractions, mutant PrP^Q227X^ forms large aggregates and recovered in the bottom fractions of sucrose gradients. The formation of PrP^Q227X^ aggregates may occur in extracellular space similar to amyloid plaques in Alzheimer’s disease since most of the protein is released from neurons as shown in previous cell and animal models [4, 12, 35] and in our current study.

Although no, or few PrP^Wt^ if present, was converted into PrP^Sc^ by the pathological mutant PrP^Q227X^ in the patient, the PrP^Q227X^-derived PrP^Sc^ from the brain homogenate of this GSS patient was able to seed conversion in vitro by PMCA in the presence of human PrP^Wt^ from Tg mice as the substrate, which is consistent with previous studies in cell-free and cell models [6–8]. However, consistent with the in vivo finding, PrP^Sc^ from GSS^Q227X^ failed to convert wild-type PrP^C^ from normal human brain homogenates although PrP^Sc^ from sCJDVV2 converted human PrP^C^-129VV to PrP^res^. The mechanism underlying the discrepancy in the convertibility of PrP^Sc^ as the seeds from this case with PrP^C^ as the substrate between humanized Tg mouse brain and autopsied normal human brain remains to be determined in the future. Our one-dimensional gel electrophoresis and Western blotting showed that the PrP^Q227X^-derived PrP^Sc^ is composed of smeared PrP bands that are expected to be a mixture of aggregates of the unglycosylated or less glycosylated mutant PrP molecules, supported by our findings using sucrose gradient sedimentation analysis. However, when PrP^Q227X^ was used to seed amplification by PMCA in the presence of PrP^Wt^ from the humanized Tg mouse brain homogenate, the gel profile of the amplified PrP^Sc^ virtually was similar to that of PrP^Sc^ amplified from sCJD controls. This phenomenon was also observed when PrP^Sc^ from VPSPr was amplified by PMCA, which also generated a PrP^res^ similar to that of sCJD controls [23]. It seems that the atypical PrP^Sc^ does not follow the template-directed refolding hypothesis instead of the seed-directed refolding hypothesis.

Our cell models expressing either human PrP^Q227X^, PrP^Wt^, or PrP^T183A^ confirmed the previous studies that the anchorless mutant PrP is less glycosylated and mainly secreted into the cell culture medium (secreted PrP) while a small portion remained associated with the cell membrane or within the cytosol (cellular PrP) [4, 12, 35]. Compared with cellular PrP^Q227X^, secreted PrP^Q227X^ exhibited additional truncated fragments (6–7 vs 2 PrP fragments). Moreover, different from secreted PrP^WM^, secreted PrP^Q227X^ notably formed extra small truncated PrP fragments. It would be interesting to discern whether the highly prone to protein truncation of anchorless PrP^Q227X^ results from the lack of the GPI anchor, the protein mutation, or both. The formation of more truncated small fragments by the anchorless protein may contribute to the intense amyloid fibril formation in the extracellular space in transgenic mice expressing anchorless PrP [12, 13]. It has been known that the generation of more small protein fragments is associated with deposition in the brain of extracellular amyloid plaques in GSS and Alzheimer’s disease.

Based on our previous 2D study [17], human brain-anchored PrP^C^ migrates at between 35 and 26 kDa, with *p*I ranging from 4.5 to 10, including full-length di-, mono-, and non-glycosylated PrP molecules and truncated glycosylated PrP fragments; after deglycosylation with PNGase F, they shifted to between 26 and 20 kDa, *p*I ranging from 6.5 to 10 representing deglycosylated full-length and truncated PrP. Interestingly, a 2D study with prion-infected transgenic mouse brain expressing mouse anchorless PrP revealed that there are two groups of PrP spots migrating at 26 kDa, with *p*I ranging from 8 to 9.2 while deglycosylated anchorless PrP migrated at 23 kDa, *p*I 9.4; they confirmed that lack of the GPI anchor induces an alkaline shift of ~ 1 *p*I unit of PrP and that anchorless PrP is characterized by immature glycosylation and altered endogenous proteolytic processing [37]. Our current study using 2D gel electrophoresis and Western blotting displayed for the first time that the *p*Is of PrP^Q227X^ differed significantly between proteins in cells vs. culture medium, in addition to the difference in the molecular weight of PrP bands shown by 1D Western blotting. While cellular PrP^Q227X^ migrated from 22 to 25 kDa with acidic *p*Is from 3.5 to 5.5, the secreted mutant protein spots recovered in the culture medium were clustered with molecular weights at 18–26 kDa at neutral to basic *p*Is from 7 to 10. Given that both cellular and secreted PrP^Q227X^ molecules were not or less glycosylated, one of the reasons that there are such different *p*Is between the two groups of mutant PrP molecules could be associated with different truncated PrP fragments. The exact molecular mechanism underlying their differences needs to be further determined in the future.

## Conclusions

In summary, our current study provides the first evidence that the PrP^Sc^ molecule in GSS linked to the PrP^Q227X^ mutation is predominately derived from the mutant PrP and it is able to convert human PrP^C^ from humanized Tg mice but not from human brain PrP^C^ in vitro. Moreover, the cellular and secreted forms of PrP^Q227X^ have significant differences in their isoelectronic points and fragment compositions, which may determine their trafficking and distribution. We believe that these findings are helpful for improving our understanding of the role of the PrP GPI anchor in human PrP formation and the pathogenesis of human prion diseases.

## Data Availability

All materials used in this study will be made available subject to a material transfer agreement.

## Abbreviations

PrP^C^: cellular prion protein
PrP^Sc^: scrapie isoform of prion protein
GSS: Gerstmann-Sträussler-Scheinker syndrome
sCJD: sporadic Creutzfeldt-Jakob disease
GPI: glycosylphosphatidylinositol
PMCA: protein misfolding cyclic amplification
Tg: transgenic
2D: two-dimensional electrophoresis
